# Should We Continue Assessing Glomerular Filtration Rate with the Cockroft–Gault Formula in NOAC-Treated Patients? The Magnitude of the Problem

**DOI:** 10.3390/jcm9061893

**Published:** 2020-06-17

**Authors:** Roberto Cemin, Luisa Foco, Carmine Zoccali, Raffaele De Caterina

**Affiliations:** 1 Department of Cardiology, San Maurizio Regional Hospital of Bolzano, 39100 Bolzano, Italy; ROBERTO.CEMIN@sabes.it; 2 Institute for Biomedicine, EURAC Research, Affiliated Institute of the University of Lubeck, 39100 Bolzano, Italy; luisa.foco@eurac.edu; 3 Unit of Clinical Epidemiology and Pathophysiology of Renal Diseases and Hypertension, Institute of Clinical Physiology (IFC)-Italian National Research Council (CNR), 89129 Reggio Calabria, Italy; carmine.zoccali@tin.it; 4 Institute of Cardiology, University of Pisa, 56126 Pisa, Italy

**Keywords:** atrial fibrillation, non-vitamin K antagonist oral anticoagulants, renal function, reclassification

## Abstract

Despite the proven superiority of the Chronic Kidney Disease Epidemiology Collaboration (CKD-EPI) over the Cockcroft–Gault (CG) formula, current guidelines recommend the latter to assess renal function in patients treated with non-vitamin K antagonist oral anticoagulants (NOACs). To assess the relationship between the CG and the recommended CKD-EPI formulas, in a cohort of atrial fibrillation (AF) patients treated with NOACs, and the misclassifications introduced by the CG formula for renal function levels, we estimated renal function with three equations: CG, CKD-EPI with body surface adjustment (1.73 mL/m^2^, CKD-EPI) and without such adjustment (CKD-EPI_noBSA), in all consecutive AF patients discharged from NOACs from the Cardiology Division of a main city hospital between February 1st and May 31st 2018. We compared the different estimates of glomerular filtration rate and potential renal function class misclassifications. We reclassified 37/115 patients (32.1%) when switching from the CG to the CKD-EPI; and 24/115 (20.8%) switching from the CG to the CKD-EPI_noBSA formulas. Class reallocation was distributed across all levels of renal function, but mostly affected the “hyper-normal” function. In estimating consequences of such reallocation, a change in NOAC dosages would have occurred in 10/115 patients (8.7%) when switching from the CG to the CKD-EPI formula and in 10/115 patients when switching from the CG to the CKD-EPI_noBSA formula. Although the CG method has been traditionally used to calculate renal function in all NOAC studies, a renal dysfunction class reallocation occurs in a substantial fraction of hospital-admitted AF patients with the use of better estimates of renal function.

## 1. Introduction

In recent years, randomized controlled trials and observational studies have been performed in patients treated with the non-vitamin K antagonist oral anticoagulants (NOACs), currently used for stroke prevention in atrial fibrillation (AF) [1,2] or the prevention and treatment of venous thromboembolism (VTE) [3,4,5]. NOACs are increasingly prescribed in daily clinical practice worldwide [6]. With the notable exception of betrixaban [7], NOACs are cleared to a significant extent by the kidneys, and renal elimination is about 80% for dabigatran, 50% for edoxaban, 35% for rivaroxaban and 27% for apixaban [8]. Therefore, the use of these drugs poses peculiar problems in patients with chronic kidney disease (CKD), a growing population that in 2017 attained the 850 million mark on a world scale [9]. Of note, CKD has a bidirectional interaction with AF: in fact, AF is a risk factor for CKD and vice versa [10,11]. A similar bidirectional link exists for deep vein thrombosis [12] and pulmonary embolism [13]. The risk for both thromboembolic and severe bleeding events is known to be high in CKD [1], and particularly so, in patients with end-stage kidney disease (ESKD) [14], making risk stratification for the prevention or treatment of thromboembolism particularly challenging in the CKD population [15,16]. Given the renal clearance problems of NOACs [8], adequate estimates of renal function are necessary to prescribe these drugs correctly. The current European Society of Cardiology (ESC) guidelines recommend the use of dabigatran only in patients with an estimated creatinine clearance (eCrCl) ≥ 30 mL/min/1.73 m^2^, and for rivaroxaban, apixaban and edoxaban in those with a CrCl > 15 mL/min/1.73 m^2^ [1]. In 2018, the European Heart Rhythm Association (EHRA) issued a warning that in patients with “hyper-normal” renal function (i.e., glomerular filtration rate (GFR) > 95 mL/min/1.73 m^2^) current dosages of edoxaban, rivaroxaban and apixaban might be insufficient [17].

In all NOAC registration studies that compared these drugs with warfarin [18,19,20,21,22], GFR was estimated through the assessment of CrCl, in turn estimated with the Cockcroft–Gault (CG) formula (CG-CrCl). However, neither this time-honored formula [23], nor its correction for the body surface area (BSA) [24] are still longer recommended by current international guidelines by the Kidney Diseases Global Outcomes (KDIGO) [25] and by the National Kidney Foundation [26]. These guidelines recommend creatinine measurements traceable to the isotopic dilution mass spectroscopy (IDMS) standard and estimates of the GFR (eGFR, Table S1) by the Chronic Kidney Disease Epidemiology Collaboration (CKD-EPI) equation, which is more accurate than the previous Modification of Diet in Renal Diseases (MDRD) formula and the CG-CrCl [27,28,29]. The CKD-EPI Creatinine Equation is normalized for BSA, calculated with the Mosteller equation [30], and if weight and height are known, the “not normalized CKD-EPI (CKD-EPI_noBSA)” version could increase the accuracy of the GFR assessment, especially in patients in the upper and lower weight ranges [31,32]. Despite the undisputed superiority of the CKD-EPI formula over the CG-CrCl, and other creatinine-based formulas, the 2018 version of the EHRA Practical Guide on NOACs [17], as well as European Society of Cardiology (ESC) [1,33] and North American consensus documents and guidelines on AF [2] still recommend the CG formula to measure renal function in patients, where treatment with NOACs is considered and in patients already on treatment. Such a recommendation depends on the fact that in the main NOAC trials the CG-CrCl (uncorrected for BSA) was adopted. If the NOAC pharmacokinetics depends on renal function—as it does—and if dose adjustments in the presence of renal dysfunction are needed to correctly prescribe the NOAC dose, more precise estimates of the GFR should provide a more reliable guide for the use of these drugs in patients with CKD.

In this context, we assessed the relationship between CG-CrCl and the recommended standard for the estimate of GFR in CKD patients, the CKD-EPI formula, in an incident series of AF patients treated with NOACs, and evaluated the potential misclassification introduced by CG-CrCl for EHRA renal function strata [17], commonly used now in cardiology and internal medicine to adjust NOAC dosing according to NOAC labels.

## 2. Materials and Methods

We assessed the renal function in all consecutive patients with AF discharged on a NOAC from the Department of Cardiology of the San Maurizio Regional Hospital of Bolzano, Italy, during the 4 month period between February 1st and May 31st 2018. Written informed consent to retrospective evaluations of the clinical data accrued during the admission was obtained from all patients. A formal approval of the protocol from the local Ethics Committee was considered unnecessary because the study did not interfere with the usual clinical routine, the assessment of renal function was part of the routine examinations prescribed to patients, no change in routine dose administration of the NOACs was implemented based on the study results, and data were handed anonymously. In all patients, plasma creatinine was measured before discharge using the recommended IDMS traceable enzymatic colorimetric method (COBAS c 111 system; Roche diagnostic GmbH, Mannheim, Germany).

CrCl was estimated by the CG [23] and the eGFR by the CKD-EPI formula, with (CKD-EPI) [34] and without body surface adjustment (CKD-Epi_noBSA) [35]. Additional analyses were also performed with the BSA adjustment of the CG formula (CG-BSA). We compared these eGFR estimates with each other, and evaluated the potential misclassification introduced by the CG-CrCl for EHRA renal function strata [17]. To this end, patients were grouped as suggested in the EHRA NOAC practical guide [17] according to their class of renal function [34] (Table S2). The misclassification by the CG-CrCl compared to the CKD-EPI and CKD-EPI_noBSA was calculated across the EHRA NOAC renal function strata.

### Statistical Analyses

We described continuous variables using range, means and standard deviations, medians and interquartile ranges. We assessed the departure from normality of the CG-CrCl, the CG-BSA, the CKD-EPI and the CKD-EPI_noBSA using the skewness and kurtosis tests for normality. We inspected the data using scatter plots, as well as adding both linear and non-linear fitting functions. We assessed the agreement between the different GFR values in the same patients through the three different equations using Lin’s concordance correlation coefficient for agreement rho (*ρ*_c_) on a continuous measure [36,37]: this is the product of the Pearson correlation coefficient and a bias factor for measuring accuracy. We generated Bland and Altman plots [38], displaying the limits of agreement of the different methods. We performed all the analyses using the Stata 16.1 software (StataCorp, College Station, TX, USA). In particular, we estimated fractional polynomials using the commands “fpfit” and “fp” regression; we estimated the agreement of the different methods using the “concord” command.

## 3. Results

**Figure 1:**
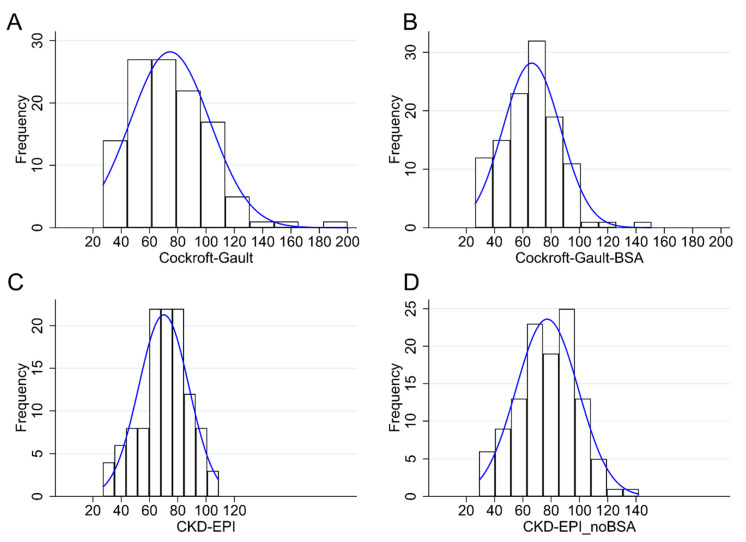
Distribution of glomerular filtration rate (GFR) measurements obtained with (**A**) the Cockcroft–Gault (CG), (**B**) the CG-BSA, (**C**) the Chronic Kidney Disease Epidemiology Collaboration (CKD-EPI) and (**D**) the CKD-EPI_noBSA formulas. BSA: body surface area.

The source population was composed of 646 patients with AF. Five hundred and thirty-one were excluded because they were not on NOAC treatment, and 115 could be eventually included. Eighty-three of these (72.2%) were male and 32 (27.8%) were female; all of them were Caucasian. Mean age was 69 years and mean plasma creatinine concentration was 1.1 mg/dL. The main demographic characteristics of the population are shown in Table 1. The NOACs used in these patients were rivaroxaban in 59 (51.3%), apixaban in 27 (23.5%), edoxaban in 15 (13.0%) and dabigatran in 14 (12.2%). Only in one case, dabigatran was prescribed even if contraindicated by a reduction of CrCl. In all the other patients, the dosages of the NOACs were appropriately adjusted to renal function. Median CG-CrCl was 75 mL/min; the GFR estimated by the CKD-EPI, the CKD-EPI_noBSA and the CG-BSA equations were 72 mL/min/1.73 m^2^, 79 mL/min and 67 mL/min/1.73 m^2^, respectively (Table 1). There was no departure from normality for the CKD-EPI and the CKD-EPI_noBSA formulas, while the distribution of CG-CrCl was skewed to the left (*p* < 0.0001; Figure 1). The distribution of the CG-BSA formula was also skewed to the left (*p* = 0.005, Figure 1).

**Figure 2:**
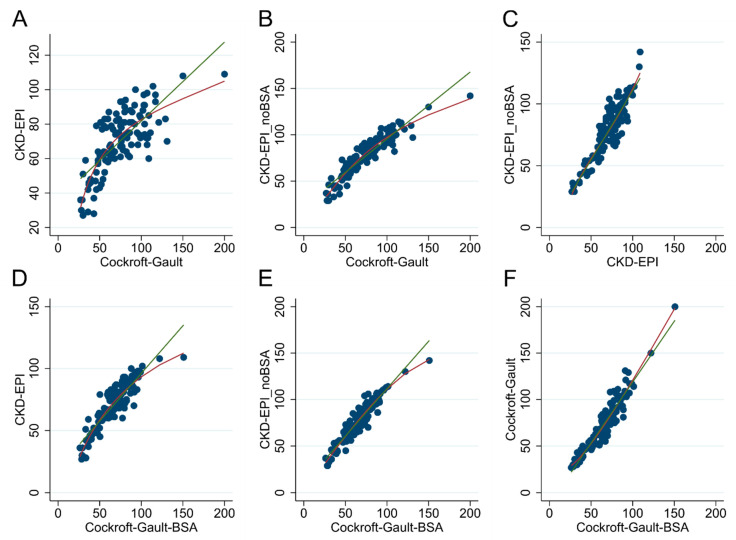
Scatter plots with fitting trend lines of glomerular filtration rate (GFR) measurement obtained with the Cockcroft–Gault (CG), the CG-BSA, the Chronic Kidney Disease Epidemiology Collaboration (CKD-EPI) and the CKD-EPI_noBSA formulas. Green: (**A**) linear prediction; red: non-linear prediction (fractional polynomial). CKD-EPI vs. CG, (**B**) CKD-EPI_noBSA vs. CG, (**C**) CKD-EPI_noBSA vs. CKD-EPI, (**D**) CKD-EPI vs. CG-BSA, (**E**) CKD-EPI_noBSA vs. CG-BSA and (**F**)CG vs. CG-BSA. The relationship between the CG and CKD-EPI is better described fitting a fractional polynomial curve, compared to a linear model (*p* < 0.0001). The same relationship applies to the CG and CKD-EPI_noBSA (*p* < 0.0001), while there is no evidence of improved fitting using fractional polynomials when comparing CKD-EPI and CKD-EPI_noBSA (*p* = 0.838). Similar relationships are observed between CG-BSA vs. CKD-EPI (*p* < 0.0001), CG-BSA vs. CKD-EPI_noBSA (*p* = 0.382), and CG-BSA vs. CG (*p* = 0.036). BSA: body surface area.

As expected, the renal function estimates by the four equations (Figure 2) were highly inter-correlated. The correlation between the CG and the CKD-EPI appeared to be less linear, and was actually better approximated by an exponential-like function (*p* < 0.0001). The same occurred for the CG-BSA correlation with the CKD-EPI (Figure 2).

As expected, Lin’s concordance correlation coefficients (*ρ*_c_) showed a good pairwise agreement between the CKD-EPI and the CKD-EPI_noBSA (*ρ*_c_ = 0.83; 95% CI: 0.78–0.88, *p* < 0.0001), while agreement was lower for the CG vs. the CKD-EPI (*ρ*_c_ = 0.65; 95% CI: 0.56–0.73, *p* < 0.0001) and higher for the CG vs. the CKD-EPI_noBSA (*ρ*_c_ = 0.89; 95% CI: 0.86–0.92, *p* < 0.0001). The agreement for the correlations with the CG-BSA with the other formulas used were: for CG-BSA and CKD-EPI (*ρ*_c_ = 0.86; 95% CI: 0.82–0.90, *p* < 0.0001); for CG-BSA and CKD-EPI_noBSA (*ρ*_c_ = 0.83; 95% CI: 0.78–0.88, *p* < 0.0001); for CG-BSA and CG (*ρ*_c_ = 0.85; 95% CI: 0.81–0.89, *p* < 0.0001).

**Figure 3:**
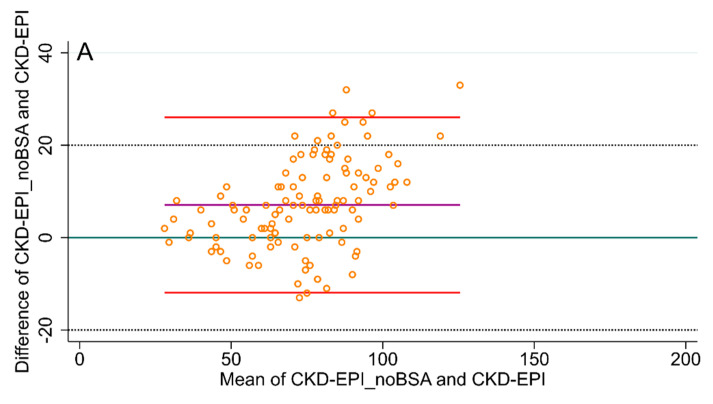

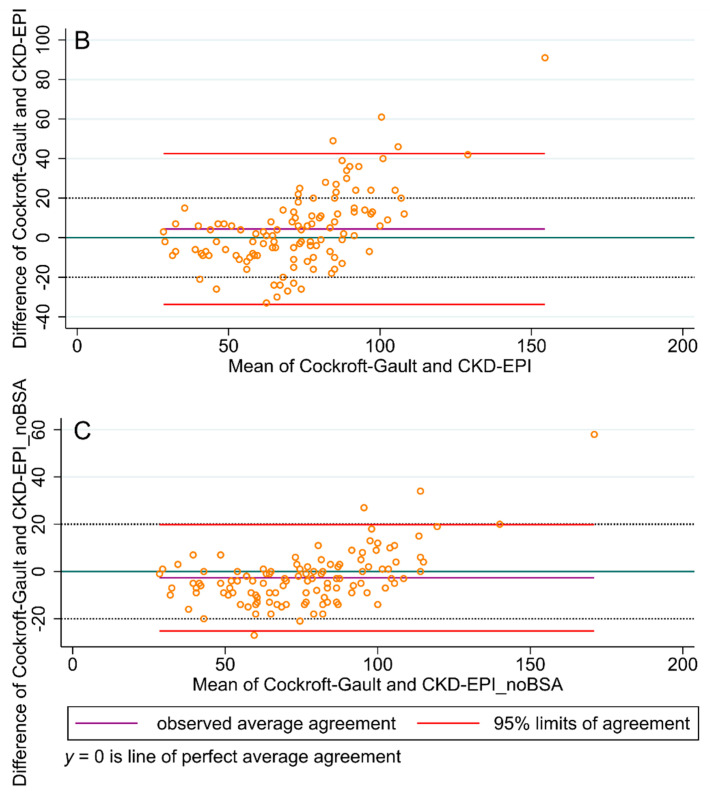
Bland and Altman plots comparing the glomerular filtration rate (GFR) measured with CKD-EPI_noBSA, as well as the Chronic Kidney Disease Epidemiology Collaboration (CKD-EPI) and Cockcroft–Gault formulas. *y* = 0 is the line of perfect average agreement. (**A**) CKD-EPI_noBSA vs. CKD-EPI, (**B**) CKD-EPI vs. Cockcroft–Gault, (**C**) CKD-EPI_noBSA vs. Cockcroft–Gault. BSA: body surface area.

**Figure 4:**
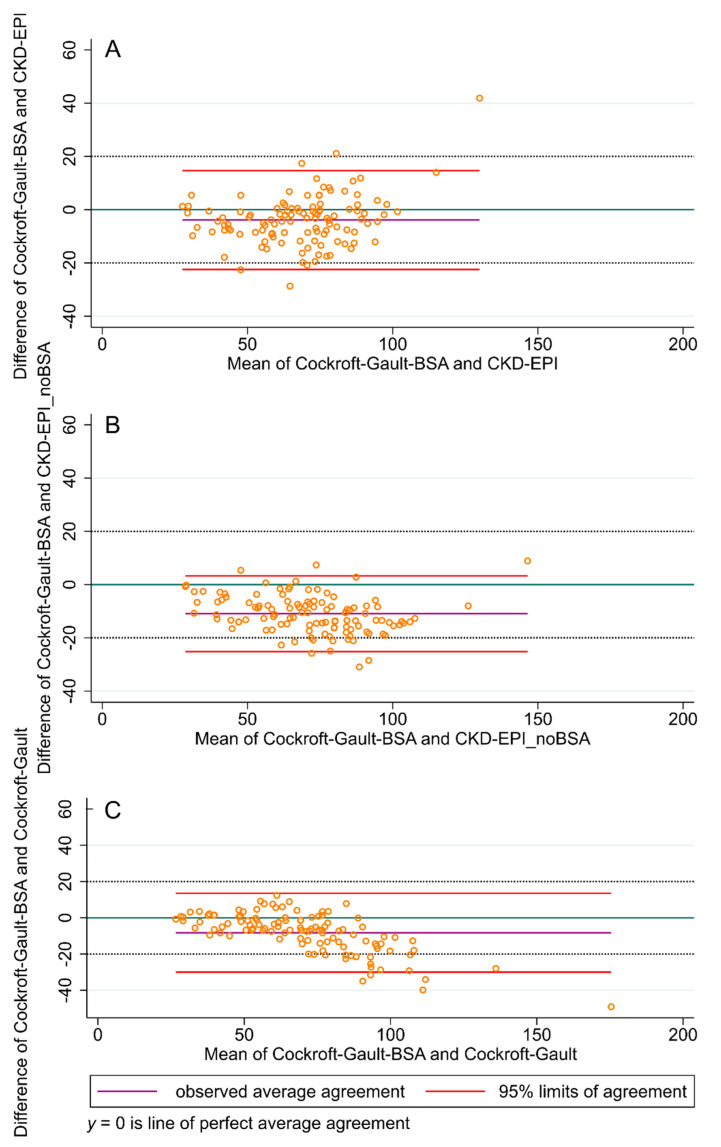
Bland and Altman plots comparing the glomerular filtration rate (GFR) measured with CKD-EPI_noBSA as well as the Chronic Kidney Disease Epidemiology Collaboration (CKD-EPI), Cockcroft–Gault and the Cockcroft–Gault-BSA formulas. *y* = 0 is the line of perfect average agreement. (**A**) CKD-EPI_noBSA vs. Cockcroft–Gault-BSA, (**B**) CKD-EPI vs. Cockcroft–Gault-BSA, (**C**) Cockcroft–Gault vs. Cockcroft–Gault -BSA. BSA: body surface area.

In the Bland and Altman plots comparing the GFR estimated with CKD-EPI, and the CKD-EPI_noBSA and CG formulas (Figure 3), just few data points were outside the 95% limits of the agreement boundaries. However, in as many as 28 patients (24.3%), the difference between the CG and the CKD-EPI values exceeded 20 mL/min, which is considered a relevant difference in GFR in clinical practice. On the other hand, only in five patients (4.3%) was the difference between CG and CKD-EPI_noBSA higher than 20 mL/min. A similar pattern occurred for the comparison of the CG-BSA, CKD-EPI and the CKD-EPI_noBSA formulas (Figure 4).

### Patient Reclassification According to CrCl, eGFR by the CKD-EPI and CKD-EPI_noBSA Equations

Using the renal function categories adopted by the EHRA guidelines, and based on CG-CrCl (Table S2), 37 patients (32.1%) were reclassified to another renal function category when switching from the CG formula to the CKD-EPI, and 24 (20.8%) when switching from the CG to the CKD-EPI_noBSA formula. This reallocation was distributed across all the renal function strata, particularly so in the “hyper-normal” function when the eGFR was estimated with the CKD-EPI formula (Table 2 and Table 3).

Regarding NOAC dosages in our population, this reallocation of renal function would have demanded a dosage change in 10 patients (8.7%) when switching from the CG formula to the CKD-EPI, and in 10 patients (8.7%) when switching from the CG formula to CKD-EPI_noBSA. The use of both CKD-EPI and CKD-EPI_noBSA would have allowed an appropriate prescription of dabigatran in the single patient in whom it had been inappropriately prescribed following the CG formula.

Data regarding reclassifications from the rarely used CG-BSA formula to CKD-EPI, to CKD-EPI_noBSA and from the CG-BSA to the CG formulas, are reported for completeness in Table 4, Table 5 and Table 6.

## 4. Discussion

We reclassified a substantial proportion (32.1% and 20.8%) of patients when switching from the still widely used CG to the CKD-EPI and to the CKD-EPI_noBSA formulas, respectively. Class reallocation was distributed across all EHRA renal function strata, but also largely affected patients with “hyper-normal” renal function. Such reallocation would have demanded a change in NOAC dosages in about 9% of the patients when switching from the CG to either the CKD-EPI or the CKD-EPI_noBSA formulas. A similar overall potential 1.5–11% change in NOAC prescription dosages has been previously described [39]. These proportions are of clinical relevance, and set a rational basis for larger studies including pertinent end-points, like bleeding or thrombosis, to compare the safety of dosing protocols based on the CG-creatinine clearance vs. the CKD-Epi eGFR to guide NOAC prescription in CKD.

The good correlation in GFR estimates of the four different methods here adopted was obvious and expected, being all formulas based on the same measurement of creatinine and using partially overlapping demographic and anthropometric variables. Useful information, conversely, can be obtained by analyzing differences in classifications according to the four methods. While GFR values by the CKD-EPI and the CKD-EPI_noBSA formulas are normally distributed, CrCl by the CG methods are skewed to the right, and feature a larger number of patients with supra-normal values as compared to the other methods (Figure 1). This goes hand-in-hand with the observation that the correlation between the CG and the CKD-EPI formulas does not appear to be linear, and is better approximated by an exponential function. Deviations in the CG formulas from the CKD-EPI formula occur at higher ranges of GFR, where the CG formula, which include body weight, are known to overestimate the real GFR in the obese [40].

The main observation in this study is that a consistent proportion of AF patients was reclassified into a different EHRA-renal function stratum when the CKD-Epi and the CKD-EPI_noBSA were applied: 32.1% when switching from the CG-Cr.Cl to CKD-EPI and 20.8% when switching the CKD-EPI_noBSA method. Within specific classes of renal function, CKD-EPI and CKD-EPI_noBSA both reclassified toward a normal renal function 44% of the patients classified as with mild or moderately depressed (Cr-Cl 30–50 mL/min) renal function estimated with the CG, confirming an underestimation of CG-CrCl in similar patients (CrCl < 60 mL/min) as previously described [39]. In addition, however, 17.3% (20/115) of our patients were reclassified from the >95 mL/min/1.73 m^2^ stratum to ≤95 mL/min/1.73 m^2^ strata by the CKD-EPI formula. For patients originally categorized as with hyper-normal renal function (i.e., CrCl > 95 mL/min) with the CG, there was a reclassification to normal in 77% and 31% of the patients when using the CKD-EPI and the CKD-EPI_noBSA, respectively. Other authors [41] have reported a lower rate of misclassification at this level (24–39%), which was reduced to 7–14%, when correction for the BSA was used. We could not confirm this, as we found, on the contrary, that reclassification to normal was higher with the CKD-EPI without BSA correction.

In the broad picture of previous literature on the topic, a comprehensive compilation, with no language limitation, of all previous studies is provided in Table S3 in the Supplementary Materials. From its examination, one gathers that reclassifications occur indeed at any level of renal function, but are clearly more prominent in classes of lower renal function, and in patient subcategories including the elderly (>75 years of age) and patients with low body weight. Such changes should mostly affect the plasma concentrations—and therefore the anticoagulant activity—of the NOACs that more largely depend on renal clearance, such as dabigatran, edoxaban and rivaroxaban. A novelty in our study was that substantial reclassification also occurred in the high range of renal function, limiting the number of cases of defined as “hyperfiltration”. Both an underestimation and an overestimation of renal function are of concern: an underestimation of renal function could translate into the prescription of an inappropriately low dosage of a NOAC, exposing patients to a higher risk of thromboembolism. Conversely, an overestimation of renal function might prompt the prescription of a higher dosage, and consequently, a higher risk for bleeding, or—at the high range—deny the benefits of the NOACs in favor of warfarin in some patients due to concerns related to the decreased anticoagulant activity of the NOACs in such cases [17,42].

The limitations of the CG formula cannot be overemphasized, and these are not circumvented with the adjustment for BSA, which is very rarely used [40]. This formula was derived from serum creatinine measurements which were uncalibrated to the IDMS standard, which is now demanded for creatinine-based estimates of the GFR by the CKD-Epi formula. The uncalibrated creatinine method used to develop the CG equation is no longer in use, and may overestimate IDMS-calibrated creatinine by 10–20%, thereby introducing a non-trivial degree of inaccuracy. In general, the risk of inaccurate estimations of renal function is relevant for drugs with a narrow therapeutic window [43], and may also be important for NOAC prescription.

### Limitations

This study has limitations. The first is the relatively small sample size, sufficient, however—we believe—to draw the main conclusions. A second—more conceptual—limitation, probably more relevant, is the assumption that a reclassification of patients at borderline values of GFR for dose reduction or increase based on a more accurate estimate of the GFR would lead to better outcomes in stroke prevention and/or bleeding. This fundamental question is still unanswered, and might be further explored in ongoing prospective non-interventional trials being run [44]. In these, one could compare the risk for thrombotic and bleeding events in patients where the application of the CKD-EPI formula would demand a dose adjustment different from that dictated by the CG formula vs. those in whom the difference between the two estimates of renal function is immaterial for drug dosing.

## 5. Conclusions

Although the CG method has been traditionally used to calculate renal function in all NOAC studies, this method is no longer considered an accurate way to assess renal function. Substantial reclassifications of patients occur across the entire spectrum of renal function, but are more clinically relevant at the lower and also—a novelty of our study—at the higher end of renal function. Research efforts are needed to test whether NOAC prescription guided by indices of renal function better than the CG formula may optimize drug plasma levels and reduce the risk for excessive or defective anticoagulation in patients with CKD. This is an area where registry data, in the absence of randomized comparisons, can provide useful information.

## Figures and Tables

**Table 1:** Demographic characteristics of the enrolled patients.

	Range	Mean (Standard Deviation)	Median (Interquartile Range)
Age (years)	43.7–95.1	69 (12.1)	69.9 (57.7–78.8)
Weight (kg)	45–124	78.1 (16.8)	75 (66–89)
Height (cm)	154–190	171.1 (8.8)	172 (164–178)
Body Mass Index (kg/cm^2^)	17.6–37.9	26.5 (4.5)	25.4 (23.7–28.7)
Creatinine (mg/dL)	0.5–2.3	1.1 (0.3)	1 (0.9–1.2)
GFR (Cockcroft–Gault) (mL/min)	29–142	77.2 (22)	75 (54–92)
GFR (CKD-EPI) (mL/min/1.73 m^2^)	27–109	70.1 (17.7)	72 (60–82)
GFR (CKD-EPI_noBSA) (mL/min)	27–200	74.5 (28.1)	79 (63–92)
GFR (Cockcroft–Gault-BSA) (1.73 mL/min)	26–151	66.3 (20.3)	67 (53–78)

GFR: glomerular filtration rat; CKD-EPI: Chronic Kidney Disease Epidemiology Collaboration; BSA: body surface area.

**Table 2:** Reclassification of patients to different groups of renal function switching from the Cockcroft–Gault formula to the CKD-EPI formula.

	CKD-EPI						% Reclassified		
Cockcroft–Gault	Hyper Normal	Normal	Mild or Moderately Depressed	Severely Depressed	End Stage	Total	To Higher GFR	To Lower GFR	Total
Hyper Normal	6 (23.08)	20 (76.92)	0 (0)	0 (0)	0 (0)	26 (100)		76.9	76.9
Normal	1 (1.47)	64 (94.12)	3 (4.41)	0 (0)	0 (0)	68 (100)	1.5	4.4	5.9
Mild or Moderately Depressed	0 (0)	7 (38.89)	8 (44.44)	3 (16.67)	0 (0)	18 (100)	38.9	16.7	55.6
Severely Depressed	0 (0)	0 (0)	3 (100)	0 (0)	0 (0)	3 (100)	100		100
End Stage	0 (0)	0 (0)	0 (0)	0 (0)	0 (0)	0 (0)			
Total	7 (6.09)	91 (79.13)	14 (12.17)	3 (2.61)	0 (0)	115 (100)			

Note: The percentage of patients reclassified has been calculated using the row totals within each group as denominator. GFR: glomerular filtration rate; CKD-EPI: Chronic Kidney Disease Epidemiology Collaboration.

**Table 3:** Reclassification of patients to different groups of renal function switching from the Cockcroft–Gault formula to the CKD-EPI_noBSA formula.

	CKD-EPI_noBSA						% Reclassified		
Cockcroft–Gault	Hyper Normal	Normal	Mild or Moderately Depressed	Severely Depressed	End Stage	Total	To Higher GFR	To Lower GFR	Total
Hyper Normal	18 (69.23)	8 (30.77)	0 (0.0)	0 (0.0)	0 (0.0)	26 (100)		30.8	30.8
Normal	4 (5.88)	63 (92.65)	1 (1.47)	0 (0.0)	0 (0.0)	68 (100)	5.9	1.5	7.4
Mild or Moderately Depressed	0 (0.0)	8 (44.44)	9 (50.00)	1 (5.56)	0 (0.0)	18 (100)	44.4	5.6	50.0
Severely Depressed	0 (0.0)	0 (0.0)	2 (66.67)	1 (33.33)	0 (0.0)	3 (100)	66.7		66.7
End Stage	0 (0.0)	0 (0.0)	0 (0.0)	0 (0.0)	0 (0.0)	115 (100)			
Total	22 (19.13)	79 (68.70)	12 (10.43)	2 (1.74)	115 (100)				

Note: The percentage of patients reclassified has been calculated using the row totals within each group as denominator. GFR: glomerular filtration rate; BSA: body surface area.

**Table 4:** Reclassification of patients to different groups of renal function switching from the Cockcroft–Gault-BSA formula to the CKD-EPI formula.

	CKD-EPI						% Reclassified		
Cockcroft–Gault-BSA	Hyper Normal	Normal	Mild or Moderately Depressed	Severely Depressed	End Stage	Total	To Higher GFR	To Lower GFR	Total
Hyper Normal	5 (83.33)	1 (16.67)	0 (0.0)	0 (0.0)	0 (0.0)	6 (100)		16.7	16.7
Normal	2 (2.38)	81 (96.43)	1 (1.19)	0 (0.0)	0 (0.0)	84 (100)	2.4	1.2	3.6
Mild or Moderately Depressed	0 (0.0)	9 (42.86)	10 (47.62)	2 (9.52)	0 (0.0)	21 (100)	42.9	9.5	52.4
Severely Depressed	0 (0.0)	0 (0.0)	3 (75.00)	1 (25.00)	0 (0.0)	4 (100)	75.0		75.0
End Stage	0 (0.0)	0 (0.0)	0 (0.0)	0 (0.0)	0 (0.0)	0 (100)			
Total	7 (6.09)	91 (79.13)	14 (12.17)	3 (2.61)	0 (0.0)	115 (100)			

CKD-EPI: Chronic Kidney Disease Epidemiology Collaboration.

**Table 5:** Reclassification of patients to different groups of renal function switching from the Cockcroft–Gault-BSA formula to the CKD-EPI_noBSA formula.

	CKD-EPI_noBSA						% Reclassified		
Cockcroft–Gault-BSA	Hyper Normal	Normal	Mild or Moderately Depressed	Severely Depressed	End Stage	Total	To Higher GFR	To Lower GFR	Total
Hyper Normal	6 (0.0)	0 (0.0)	0 (0.0)	0 (0.0)	0 (0.0)	6 (100)		0	0
Normal	16 (19.05)	67 (79.76)	1 (1.19)	0 (0.0)	0 (0.0)	84 (100)	19.0	1.2	20.2
Mild or Moderately Depressed	0 (0.0)	12 (57.14)	9 (42.86)	0 (0.0)	0 (0.0)	21 (100)	57.1	0	57.1
Severely Depressed	0 (0.0)	0 (0.0)	2 (50.00)	2 (50.00)	0 (0.0)	4 (100)	50.0	0	50.0
End Stage	0 (0.0)	0 (0.0)	0 (0.0)	0 (0.0)	0 (0.0)	0 (100)			
Total	22 (19.13)	79 (68.70)	12 (10.43)	2 (1.74)	0 (0.0)	115 (100)			

BSA: body surface area.

**Table 6:** Reclassification of patients to different groups of renal function switching from the Cockcroft–Gault-BSA formula to the Cockcroft–Gault formula.

	Cockcroft–Gault						% Reclassified		
Cockcroft–Gault-BSA	Hyper Normal	Normal	Mild or Moderately Depressed	Severely Depressed	End Stage	Total	To Higher GFR	To Lower GFR	Total
Hyper Normal	6 (100)	0 (0.0)	0 (0.0)	0 (0.0)	0 (0.0)	6 (100)		0	0
Normal	20 (23.81)	62 (73.81)	2 (2.38)	0 (0.0)	0 (0.0)	84 (100)	23.8%	2.4%	26.2%
Mild or Moderately Depressed	0 (0.0)	6 (28.57)	15 (71.43)	0 (0.0)	0 (0.0)	21 (100)	28.6%	0	28.6%
Severely Depressed	0 (0.0)	0 (0.0)	1 (25.00)	3 (75.00)	0 (0.0)	4 (100)	25.0%		25.0%
End Stage	0 (0.0)	0 (0.0)	0 (0.0)	0 (0.0)	0 (0.0)	0 (100)			
Total	26 (22.61)	68 (59.13)	18 (15.65)	3 (2.61)	0 (0.0)	115 (100)			

BSA: body surface area.

## Supplementary Materials

The following are available online at https://www.mdpi.com/2077-0383/9/6/1893/s1, Table S1: Commonly adopted formulas to estimate glomerular filtration rate Cockroft–Gault formula, as used in this study, Table S2: EHRA NOAC renal function strata: classification of renal function according to GFR expressed in mL/min/1.73 m^2^ (Steffel et al. Eur Heart J 2018; 39:1330–39), Table S3: A comparation of studies addressing the classification of patients in classes of renal function with different formulae.
