# Supplementary material for: Should We Continue Assessing Glomerular Filtration Rate with the Cockroft–Gault Formula in NOAC-Treated Patients? The Magnitude of the Problem
Source: J Clin Med. 2020 Jun 17;9(6):1893. doi: 10.3390/jcm9061893 (PMC7355413; doi:10.3390/jcm9061893)
Supplement: Supplementary file 1 [file jcm-09-01893-s001.pdf]

**Supplementary material**

**Table S1.** Commonly adopted formulas to estimate glomerular filtration rate.

**Cockroft-Gault formula, as used in this study**

$$\text{CrCl [mg/dl]} = \frac{(140 - \text{age}) \times \text{weight (in kg)} \times [0.85 \text{ if female}]}{72 \times \text{SCr (in mg/dl)}}$$

**Cc**[illegible]

CrCl is creatinine clearance; SCr is serum creatinine, and age is expressed in years

**CKD-EPI**

$$\text{eGFR} = 141 \times \min\left(\frac{\text{SCr}}{\text{k}}, 1\right)^{a} \times \max\left(\frac{\text{SCr}}{\text{k}}, 1\right)^{-1.209} \times 0.993^{\text{age}} \times [1.018 \text{ if female}] \times [1.159 \text{ if black}]$$

eGFR is estimated glomerular filtration rate. SCr is serum creatinine (mg/dL), k is 0.7 for females and 0.9 for males, a is -0.329 [illegible] l, and age

$$\text{eGFR} = \frac{\text{CKD-EPI} \times 0.007184 \times \text{height (in cm)}^{0.725} \times \text{weight (in kg)}^{0.425}}{1.73}$$

eGFR is estimated glomerular filtration rate.

**Table S2.** EHRA NOAC renal function strata: classification of renal function according to GFR expressed in mL/min/1.73m$^2$ (Steffel et al. Eur Heart J 2018; 39: 1330-39).

| **GFR > 95** | **Group 1 ("hyper normal")** |
|---|---|
| 50 ≤ GFR ≤ 95 | Group 2 ("normal") |
| 30 ≤ GFR < 50 | Group 3 ("mild or moderately depressed") |
| 15 < GFR < 30 | Group 4 ("severely depressed") |
| GFR ≤ 15 | Group 5 ("end stage") |

Abbreviations: EHRA: European Heart and Rhythm Association; NOAC: non-vitamin K antagonist oral anticoagulants; GFR: glomerular filtration rate.

**Supplemental Table 3: A compilation of studies addressing the classification of patients in classes of renal function with different formulae**

| Reference | Objective | Patients | Detailed Results | Main Conclusion |
|---|---|---|---|---|
| **1** | Comparison of different estimations of renal function (CG formula vs MDRD and CKD-EPI) in patients with AF | 185 patients on NOAC for stroke prevention in AF | When CG-ClCr <70 mL/min, MDRD and CKD-EPI overestimated renal function; when CG-ClCr>70 mL/min, MDRD and CKD-EPI underestimated it | 46-50% of the patients would have received dosages inconsistent with guidelines when using MDRD and CKD-EPI instead of CG-CrCl |
| **2** | Comparison of different estimations of renal function (CG formula vs MDRD and | 3609 patients on NOAC for stroke | Among undeweight (<65 kg) and elderly (>80 years) patients, CG underestimated renal funtion compared with MDRD and CKD-EPI. CG overestimated it in overweight patients | 18-20% of on label indication for reduced dose with CG was recategorised as off label using MDRD and CKD-EPI |
| **3** | Comparison of different estimations of renal function (CG formula vs MDRD and CKD-EPI) in patients with AF | 454 patients on NOAC for stroke prevention in AF | Discrepancies were limited to patients with CG-ClCr<60 mL/min and were more evident in ≥75 years where MDRD and CKD-EPI overestimated renal function | 1-8.5% discrepancies in NOACs' dosages prescription when comparing MDRD and CKD-EPI to CG-ClCr |
| **4** | Comparison of different estimations of renal function (CG formula vs MDRD and CKD-EPI) in patients with AF and moderate chronic kidney disease | 831 patients with non-dyalisis dependent chronic kidney disease eligible for NOAC | If compared to CG-CrCl, dosages would have been reduced in 27 and 29% of the patients respectively when using MDRD and CKD-EPI formulae;<br>overtreatment would have been choosen in 9 and 7% of the patients respectively when using MDRD and CKD-EPI | 36% of the patients would have been differently classified with both MDRD and CKD-EPI if compared to CG-CrCl |
| **5** | Comparison of different estimations of renal function in patients with AF (CG | 402 non valvular AF patients on | Important changes in renal function estimates were more frequent in patients aged ≥ 75 years and a lower BMI (expecially when MDRD and CKD-EPI were used); in severly | 12-17% of the patients would change class of renal failure, using eGFR instead of eCrCl (17% with MDRD, 12% with BIS-I, 15% using CKD- |
| **6** | Comparison of different estimations of renal function (CG formula vs MDRD and CKD-EPI) in patients with AF | 910 AF patients with indication for oral anticoagulation (dabigatran, apixaban, rivaroxaban) | Discrepancies in estimation of renal function are augmented if: CG-ClCr <60 mL/min: (MDRD and CKD-EPI reclassify, respectively: 30% and 27% of patients on dabigatran; 26% and 22% of patients on rivaroxaban; 3.8% and 4.1% of patients on apixaban ≥75 years: MDRD and CKD-EPI reclassify, respectively: 18% and 15% of patients on dabigatran; 16% and 13% of patients on rivaroxaban; <5% of patients on apixaban | MDRD and CKD-EPI overestimate the renal function when compared to CG When compared to CG-ClCr, MDRD and CKD-EPI would lead to a different dosage prescription in 11.5 and 10% respectively for dabigatran, 10 and 8% for rivaroxaban, 1.4 and 1.5% for apixaban |
| **7** | Comparison of different estimations of renal function (CG formula vs MDRD and CKD-EPI). Not specific for AF patients or anticoagulant therapy | 4687 patients of NHANES database and a consecutive number of 208 patients enrolled in research studies | In patients with CG-CrCl <50 mL/min: 28-56% discrepancies in classification using MDRD and CKD-EPI; in these patients correction for BSA did note reduce misclassification. In patients with CG-CrCl >95 mL/min: 24-39% discrepancies in classification with MDRD and CKD-EPI; in these patients correction for BSA reduced misclassification to 7-14%. | Individuals who weighed less had higher eGFR than CK-CrCl and vice versa. In older adults (>65 y), eGFR were higher than CK-CrCl estimates and vice versa.<br>At lower clearences (CG-CrCl <50 mL/min and <30 mL/min) eGFR overestimated renal function (28-56% respectively). |
| **8** | | Commentary to Malavasi et al. | | |
| **Current study** | Comparison of different estimations of renal function (CG formula vs CKD-EPI and CKD-EPI_noBSA) in patients with AF | 115 patients on NOAC for stroke prevention in AF | Comparison of different estimations of renal function (CG formula vs CKD-EPI and CKD-EPI_noBSA) in patients with AF | Comparison of different estimations of renal function (CG formula vs CKD-EPI and CKD-EPI_noBSA) in patients with AF |

**References (to papers in Table S3)**


1. Kruger PC, Robinson MA, Xu K, et al. Assessing renal function in patients receiving DOACs: Cockcroft-Gault versus estimated glomerular filtration rate. Thromb Res 2017; 157: 165-6.
2. Lee KN, Choi JI, Kim YG, et al. Comparison of Renal Function Estimation Formulae for Dosing Direct Oral Anticoagulants in Patients with Atrial Fibrillation. J Clin Med 2019; 8.
3. Cabeza A, Capote P, Correa J, et al. Discrepancies between the use of MDRD-4 IDMS and CKD-EPI equations, instead of the Cockcroft–Gault equation, in the determination of the dosage of direct oral anticoagulants in patients with non-valvular atrial fibrillation. Medicina Clínica (English Edition) 2017; 150.
4. Andrade JG, Hawkins NM, Fordyce CB, et al. Variability in Non-Vitamin K Antagonist Oral Anticoagulants Dose Adjustment in Atrial Fibrillation Patients With Renal Dysfunction: The Influence of Renal Function Estimation Formulae. Can J Cardiol 2018; 34: 1010-8.
5. Malavasi VL, Pettorelli D, Fantecchi E, et al. Variations in clinical management of non-vitamin K antagonist oral anticoagulants in patients with atrial fibrillation according to different equations for estimating renal function : Post hoc analysis of a prospective cohort. Intern Emerg Med 2018; 13: 1059-67.
6. Manzano-Fernandez S, Andreu-Cayuelas JM, Marin F, et al. Comparison of estimated glomerular filtration rate equations for dosing new oral anticoagulants in patients with atrial fibrillation. Rev Esp Cardiol (Engl Ed) 2015; 68: 497-504.
7. Schwartz JB. Potential Effect of Substituting Estimated Glomerular Filtration Rate for Estimated Creatinine Clearance for Dosing of Direct Oral Anticoagulants. J Am Geriatr Soc 2016; 64: 1996-2002.
8. Riva N, Ageno W, Gatt A. Estimating renal function in patients with atrial fibrillation: which dose of direct oral anticoagulants? Intern Emerg Med 2018; 13: 1001-4.
